# Hypertension in the Neonatal Intensive Care Unit (NICU): A Case of Mid-Aortic Syndrome

**DOI:** 10.7759/cureus.35282

**Published:** 2023-02-21

**Authors:** Ayesha Ropri, Jess Randall

**Affiliations:** 1 Internal Medicine/Pediatrics, Albany Medical Center, Albany, USA; 2 Pediatrics, Albany Medical Center, Albany, USA

**Keywords:** high-output heart failure, mid aortic syndrome, newborn hypertension, renal artery stenosis, renovascular hypertension, abdominal aorta, resistent hypertension

## Abstract

A term baby was born with findings of edema, harsh murmur, and hypertension. Pregnancy course was complicated by hydrops fetalis. Upon birth, blood work did not reveal any abnormalities, but an echocardiogram showed patient in high-output heart failure. A computed tomography (CT) chest, abdomen, and pelvis revealed narrowing of aorta in the thoracic region to distal iliac and renal arteries, consistent with mid-aortic syndrome. Mid-aortic syndrome, which results in the narrowing of thoracic or abdominal aorta, is a rare cause of hypertension, especially in newborns. This case elucidates the importance of maintaining a broad differential when encountering an uncommon problem in a newborn.

## Introduction

Hypertension is a common adult problem, but the prevalence among children and adolescents is reported to be around 3.5%. Primary hypertension is reported to be the most prevalent type of hypertension diagnosed in this population [1]. In fact, guidelines suggest that children greater than six years of age, that have a positive family history of hypertension, are overweight/obese, and/or have no other clinical examination findings, do not require any further evaluation prior to a diagnosis. However, further evaluation, specifically with renal ultrasound, is recommended in patients who are less than six years of age, as renal/renovascular disease is the most common cause of secondary hypertension in this age group. Hypertension is an even more rare finding in the neonatal population, with an incidence of 0.2-0.3% [2]. Among children, renal structural anomalies account for 34-79% of hypertension, whereas renovascular disease specifically accounts for roughly 12-13% [3]. The mean age of diagnosis in studies has been noted to be around pre-teen to early-teen years, with rarely any cases diagnosed at birth [4].

Mid-aortic syndrome (MAS) is a sub-type of renovascular hypertension that is relatively uncommon. It is caused by stenosis of thoracic or abdominal aorta and/or its branches. Up to 64% of the cases are idiopathic, but some may be related to genetic disorders, such as neurofibromatosis, Alagille syndrome, or William syndrome. The most common presenting symptom is hypertension [5]. The mean age at presentation for patients is reported 9.1 (SD±5) years. Thus, it is uncommon for this rare disease to be present at birth. However, there have been three reported cases of MAS in preterm infants, one as early as 27 weeks gestational age [6,7].

## Case presentation

The patient was a 3730 g appropriate for gestational age (AGA) male neonate born to a 30-year-old G5P2022 mother at 37 1/7 weeks gestational age. Prenatal care was sought in the second trimester, and maternal history was remarkable for tobacco use, prior opioid use, and pregnancy complicated by fetal hydrops. Post-delivery management of the infant included oxygen support with continuous positive airway pressure (CPAP). Appearance, pulse, grimace, activity, and respiration (APGAR) scores were 1 at 1 minute of life, 7 at 5 minutes of life, and 9 at 10 minutes of life. Patient was subsequently transferred to the neonatal intensive care unit (NICU) due to continued need for respiratory support on CPAP.

Upon arrival at the NICU, patient was noted to have anasarca and a holosystolic murmur. He was hypertensive with blood pressure readings in the 110s/70s mmHg range that peaked at 131/91 mmHg over the next two days. Initial blood work, including complete blood count, bilirubin levels, and chemistry panel was unremarkable. Renal ultrasound (US) showed moderately echogenic kidneys with markedly elevated left main renal artery velocities. Echocardiogram revealed mild-to-moderate mitral insufficiency, mild left ventricular hypertrophy, normal left ventricular systolic function, and questionable stenosis of the celiac trunk.

**Figure 1:**
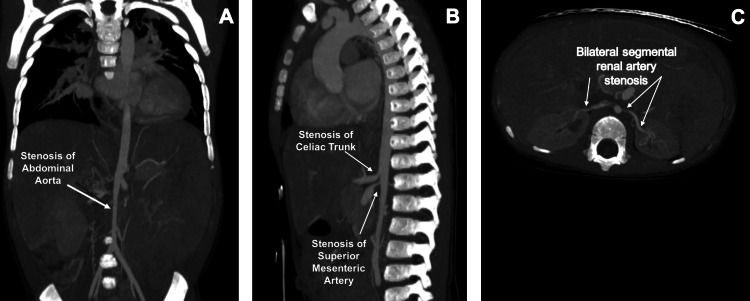
CT angiography of the chest, abdomen, and pelvis. The images show (A) coronal view of stenosis of the abdominal aorta, (B) sagittal view of stenosis at the origin of the celiac trunk and superior mesenteric artery with marked post-stenotic dilatation, and (C) axial view of bilateral segmental renal artery stenosis.

This patient presented with a clinical picture of volume overload. Fetal hydrops and anasarca can result from a range of pathophysiological factors. These include infections, chromosomal abnormalities, cardiac causes, hematologic causes, structural abnormalities, tumors, or metabolic diseases. In this case, the physical examination and initial lab work lowered infection, hematologic causes, chromosomal abnormalities, and metabolic disease lower on the differential. Further imaging, including the renal US and echocardiogram, began to reveal a possible structural abnormality. Eventually, a computed tomography (CT) scan and angiography of the chest, abdomen, and pelvis were performed, showing narrowing of the aorta in the thoracic region to distal iliac and renal arteries, as well as stenosis of the celiac trunk and superior mesenteric artery (Figures 1A-1C).

The patient was diagnosed with mid-aortic syndrome with narrowing of the aorta that was causing clinical hypervolemia and renovascular hypertension. During this time, the patient was started on medical management for blood pressure and hypervolemia.

## Discussion

Mid-aortic syndrome (MAS) is a rare cause of hypertension, especially in the newborn population. It accounts for roughly 0.5-2% of all cases of aortic stenosis in children and mean presenting age is nine years [5]. Those presenting at less than one year of age were found to have more severe disease [8]. The most common presenting symptom is uncontrolled hypertension, reported in about 87% of cases [5]. The etiology of MAS is still unknown, although it is associated with certain genetic disorders in some cases. These include William syndrome, Alagille syndrome, and neurofibromatosis type I [5]. Diagnosis generally involves imaging with a CT angiography, however, ultrasound may be used as an initial imaging modality [9]. The most common anatomic arterial site affected by MAS is suprarenal, followed by intrarenal and infrarenal branches. Renal arteries have been identified as one of the most frequent branches involved in MAS, followed by celiac artery and superior mesenteric artery [8].

Medical management is geared toward adequate blood pressure control to prevent sequelae and to preserve end-organ function. Long-term sequelae of MAS include stroke, hypertensive encephalopathy, left-ventricular dysfunction, and hypertrophy leading to heart failure and renal dysfunction [8,10]. Often, medical intervention alone is not sufficient and invasive measures are required to obtain adequate blood pressure control and prevent end-organ damage. Invasive treatment options include endovascular or surgical intervention, including aortoaortic bypass grafting, graft vascular replacement, patch angioplasty, or renal autotransplantation [5,8,10]. Both surgical intervention and endovascular repair come with risks. Long-term effects of these interventions have not been extensively studied. However, there are some studies that assessed overall outcomes for children with MAS. A single-centered retrospective study of 36 children with angiographic evidence of abdominal MAS over 30 years showed that mortality rate was 8% in that population after a median follow-up period of 4.5 years [10]. Of the children who survived, 90% had appropriately controlled blood pressure and 76% had normal estimated glomerular filtration rate (eGFR). Only six of the children in the study were managed with medication only. The study supported better outcomes for patients who had surgery, although this can be delayed with good blood pressure management [10]. Other studies describe promising results with balloon angioplasty or stenting, although complications, such as technical failures (that required re-intervention), dissections, aneurysms, and restenoses, have been reported [5,8]. More novel techniques include mesenteric artery growth improves circulation (MAGIC) and tissue expander-stimulated lengthening of arteries (TESLA) [11,12]. With MAGIC, mesenteric arteries are used to bypass the aorta as opposed to prosthetic graft material. On the other hand, TESLA utilizes the stretch-induced growth phenomenon to help vascular tissue grow slowly after placement of a tissue-expanding device. Both procedures have demonstrated more promising results in a short-term study that evaluated patients after a median of 2.5 years in conjunction with a median of one anti-hypertensive medication [12]. Of 37 patients evaluated in the study, 86.1% had normal blood pressure at the end of the study period. Further long-term studies are still needed to determine complications and maintenance of normal blood pressure.

## Conclusions

Although hypertension is one of the most common chronic conditions diagnosed in adults, it is much less common in children. Mid-aortic syndrome is a rare condition in which there is narrowing of the thoracic and/or abdominal aorta resulting in stenosis of the renal arteries. Most children are diagnosed in their adolescent stage, with relatively few cases diagnosed in infancy. Thus, it is prudent to consider a broad differential when encountering this in the neonatal setting.
